# The Lesser Omentum

**Published:** 1895-04

**Authors:** Byron Robinson

**Affiliations:** 34 Washington Street; Chicago


### THE LESSER OMENTUM.

(*Ligamentum Gastro-hepaticum, Mesogaster Anticum, Anterior Mesogaster, Mesenterium Hepatis.*)

By BYRON ROBINSON, Chicago.

The ligamentum gastro-hepaticum (lesser omentum) is a duplicature of peritoneum stretching from the lesser curvature of the stomach to the transverse fissure of the liver. It may be of interest and surprise to many to learn that the literature concerning the gastro-hepatic omentum is not only limited, but of little value. I have read Gray, Quain, Morris, Aeby and Hyrtl and many others, yet only a bare statement of origin and insertion can be found in those anatomies with the idea that it is a double membrane. Even the historical J. Mueller only writes this following short sentence (in German, in 1830,) in regard to the lesser omentum: "On the upper part of the smaller curvature the blades again meet and form a united fold reaching to the liver." This is rather a short note for a German anatomist. The ever memorable Huschke said in 1844. "The lesser omentum is the result of the separation of the liver from its embryonic connection with the stomach and is the anterior right and upper, while the gastro-splenic ligament is the greater omentum, and the posterior, left and lower." This comparison of spleen and liver as regards omenta is of little value, and I rather think misleading. The spleen and liver do not develop similarly.

Kolliker says the lesser omentum is a true liver mesentery. The master of anatomy, Henle, passes over the lesser omentum with very few words, but he gives an excellent drawing of the same in the second volume of his anatomy, page 899. From the above authors it may be said that the literature relating to the ligamentum gastro-hepaticum is quite barren. Toldt, formerly of Prague, now of Vienna, is the only one I am aware of who has studied the lesser omentum to any considerable extent. However, Toldt's excellent remarks are unfortunately very short, covering less than two pages. My studies of the lesser omentum were carried on with embryos of humans and animals, children, adult humans, and several species of adult animals. If one will study the lesser omentum on spare cadavers it will be very plain to observe that the ligamentum gastro-hepaticum is composed of three distinct parts. The lower ribs should be removed and sufficient amount of the right and left liver lobes to secure ample room to expose the whole anterior surface of the ligament as it stretches between the lower gastric curvature and the transverse fissure of the liver. I shall divide the ligamentum hepato-gastricum, or lesser omentum, into three parts for convenience of description and also to call attention to its difference of construction. The first or upper portion I shall call the pars tendineus; the second or middle portion may be named the pars flaccida (Toldt); the third or lower part will be called pars hepato-duodenale.

(1.) Pars tendineus (Robinson) is the first or upper portion. It is known by its white dense appearance. In adults, so far as my measurements are concerned, it averages two inches from top to bottom. The pars tendineus stretches from the esophagus to the transverse fissure of the liver. It loses itself in the coronary ligaments of the liver and also in the suspensory ligaments of the liver. The surface of the pars tendineus is covered anterior and posterior by a shining, serous peritoneal layer. I could separate this serous layer from both the anterior and posterior surface quite easily. Between the two thin serous layers lies the membrana mesenterii propria, or a very similar structure. The structure consists of dense, white connective tissue closely woven together. Its lower edge, reaching two inches below the diaphragm, along the right border of the esophagus is sharply defined and of a concave outline. The pars tendineus not only inserts itself into the esophagus, but many of its fibers are inserted into the cardiac part of the stomach. As it passes toward the liver it apparently passes to the transverse fissure of the liver, but its real insertion is in dense tissue surrounding the inferior hepatic vein-ligamentum venosum. It must not be forgotten that the liver is really held in position by the connection to the inferior vena cava. All other ligaments may be cut away, yet the ligamentum venosum will retain the liver in its position. Hence, this will explain why the pars tendineus has its real insertion in the ligamentum venosum. The pars tendineus is also a passage-way for blood-vessels, lymphatics and nerves to the liver. The gastric artery and the vagus nerves can easily find routes to reach the liver by way of the pars tendineus. It can be plainly observed on a spare adult cadaver that the upper portion of the lesser omentum, the pars tendineus, merges into the coronary and suspensory ligaments of the liver. The pars tendineus is recognized by its milk-white color, its tendinous and glistening appearance. It is a dense, solid connective tissue structure, situated between the right border of the esophagus and the transverse liver fissure and at the upper part of the lesser omentum. It consists of three lamina or plates. The central one (mesenterii membrana propria) is the chief one for bulk and strength. Its origin is the right esophageal border and its insertion is in the ligamentum venosum on the connective tissue surrounding the inferior vena cava. The pars tendineus, about two inches perpendicularly, is covered or faced on both sides by a very thin, shining serous layer of epithelial cells. I could easily separate both serous layers from the middle one. The lower border is concave downward and shapely defined in outline. In my examinations of fetuses the pars tendineus is well formed by the fourth month, but more and more distinct through the remaining fetal months. One can often observe this structure much earlier in some fetuses. After the sixth month of fetal life, when all essential peritoneal structures are completed, the pars tendineus is fairly plain. But, in my experience, the pars tendineus is most distinct in spare children. In children, the upper portion of the lesser omentum is very white, dense, distinct in outline and very strong. After adult years (40) the structure does not show such a plain and definite outline and some portions may be absorbed, producing a fenestrated condition. Fat accumulates in this structure very sparingly. Toldt mentions that he has observed apertures, due to absorption, in the upper portion of the lesser omentum, but I have noted this structure from fetuses up to old age and have never yet observed atrophic apertures in this structure.

(2). The pars flaccida (Toldt), or middle portion of the lesser omentum, is the next division for consideration. This part could be called very appropriately the ligamentum gastro-hepaticum. However, the structure itself has not the slightest resemblance to a ligament. It is one of the thinest double folds of peritoneum in the body. The origin of the pars flaccida is the lesser curvature of the stomach. It is simply a union of the anterior and posterior blades of peritoneum as they pass to the right from the stomach. The insertion of the pars flaccida is into the transverse fissure of the liver and the ligamentum hepatico-duodenale. Its origin is the lesser curvature of the stomach. The upper border of the pars flaccida merges into the pars tendineus by simply allowing its serous blades to divide, whence they then cover the pars tendineus on the anterior and posterior surface. At the lower border of the pars flaccida its two blades also divide and cover the anterior and posterior surfaces of the ligamentum hepato-duodenale. This portion of the lesser omentum is called pars flaccida on account of its extreme lax appearance and loose folds. It is as transparent as glass and delicate as a cob web. The apparent cause of this excessive fold is the spigel lobe of the liver. In fetuses this factor is much plainer than in adult life. Spigel's lobe is quite large, and in very early fetal life, and also the forcing downward of the stomach by the liver, as well as the distension and contraction of the stomach, keeps up a stretching of the double-bladed membrane. Motion and pressure are the causes of the pars flaccida being so abundant in its surface. It may be noted that the gastric artery never divides into arcs or arcades in the pars tendineus and flaccida (lesser omentum) as the arteries do in the great omentum. Fat is very scarce in the pars flaccida and towards its center the double-layered membrane becomes very thin. In two cadavers I have found an aperture in the pars flaccida. The apertures had rounded, smooth borders, and would admit the index finger. The pars flaccida is the anterior boundary of the antrium bursa omentalis or bursa omentalis minor. The space it encloses easily holds a large turkey egg in an adult. The space contains a viscus—Spigel's lobe.

(3.) The pars hepato-duodenale is the third and lowest portion of the lesser omentum. The origin of the pars hepato-duodenale is the upper portion of the duodenum and lower end of the stomach. Its insertion is the transverse fissure of the liver or rather in the ligamentum venosum. It merges into the pars tendineus, as one can easily prove by pulling on it, whence its insertion will show itself. The upper border of the pars hepato-duodenale is the lower border of the pars flaccida, while its lower border is a part of the circumference of the hiatus winslowii. The pars hepato-duodenale contains the hepatic artery, the hepatic duct, the portal vein, lymphatics and bundles of the sympathetic nerves, the whole being bound together by connective tissue and covered by a thin, serous layer of epithelium. This ligament forms the anterior boundary of Winslow's foramen, which generally admits two fingers in adults. It is a strong, thick ligament. The upper border of the pars hepato-duodenale is generally somewhat sharply defined, but its lower border is broad and smooth, surrounding a part of Winslow's foramen. To see the lesser omentum in an autopsy, lift up the liver and draw down the stomach, whence the three distinct portions of the lesser omentum can be viewed. The ligamentum hepato-gastricum is the anterior mesogaster. It is the double-layered membrane in which the liver developed in very early embryonic life. The irregular atrophy of the liver has directed this perineal duplicature to the right. It originally was in the mid-anterior region. A remnant of the mesogaster anticum is the ligamentum, suspensorium hepatis. One can prove it by tracing the suspensory ligament to the transverse fissure. The suspensory ligament arises from the shrinkage of the liver, for, in fetuses, the liver reaches with its umbilical fissure directly to the navel, while its lobes (right and left) reach down to the pelvis. The lesser omentum is the true mesentery of the liver. The prolapse and dilatation of the stomach is partly due to the elongation of the esophagus, but also to the elongation of the lesser omentum, especially the pars tendineus. The lesser omentum is as noted for want of fat as the great omentum is for its possession. The lower end of the lesser omentum forms part of the boundary of the foramen of Winslow. This aperture has received several names. In English it is known as the foramen of Winslow. In Latin the following appellations may be found in reference to it: the hiatus Winslowii, foramen Winslowii, orificum epiploicum Winslowii, or porta omentorum. This foramen is an oval aperture. It is bounded in front by the lower edge of the ligamentum hepato-duodenale (containing the hepatic artery, portal vein and hepatic duct); behind, by the inferior vena cava; below, by the duodenum and hepatic artery, curving forward from the coeliac axis; above, by Spigel's liver lobe and the transverse fissure of the liver. Winslow's foramen leads into an ante-room containing one viscus, viz.: Spigel's liver lobe. This ante-room or bursa omentalis minorisor antrum bursa omentalis is partially marked off from the whole lesser omental cavity by the fold of peritoneum projected up by the gastric artery and vein. This peritoneal duplicature projected forward by blood-vessels is known as the ligamentum gastro-pancreaticum. The remainder of the lesser omental sac contains no viscus and is known as the bursa omentalis majoris.

34 Washington Street.

---

Tincture of iodine of double strength, or one drachm to the ounce of 95 per cent. alcohol, when thoroughly applied by means of a feather, or better by a camel's hair pencil, to boils, etc., will relieve all pain and shorten the stages of suppuration more than one half. *Am. Med. Compend.*

